# Occurrence and Timing of Advance Care Planning in Persons With Dementia in General Practice: Analysis of Linked Electronic Health Records and Administrative Data

**DOI:** 10.3389/fpubh.2022.653174

**Published:** 2022-03-22

**Authors:** Bahar Azizi, Bram Tilburgs, Hein P. J. van Hout, Iris van der Heide, Robert A. Verheij, Wilco P. Achterberg, Jenny T. van der Steen, Karlijn J. Joling

**Affiliations:** ^1^Department of Public Health and Primary Care, Leiden University Medical Center, Leiden, Netherlands; ^2^Department of Intensive Care Medicine, Radboud University Medical Center, Radboud Institute for Health Science, Nijmegen, Netherlands; ^3^Department of General Practice and Elderly Care Medicine, Amsterdam UMC, Vrije Universiteit Amsterdam, Amsterdam Public Health Research Institute, Amsterdam, Netherlands; ^4^Nivel, Netherlands Institute for Health Services Research, Utrecht, Netherlands; ^5^Tilburg School of Social and Behavioral Sciences (TRANZO), Tilburg University, Tilburg, Netherlands; ^6^Department of Primary and Community Care, Radboud University Medical Center, Nijmegen, Netherlands; ^7^Department of Medicine for Older People, Amsterdam Public Health Research Institute, Amsterdam UMC, Vrije Universiteit Amsterdam, Amsterdam, Netherlands

**Keywords:** advance care planning, general practice, data linkage, dementia, primary care

## Abstract

**Background:**

Advance care planning (ACP) is a process of communication in which patients and family caregivers discuss preferences for future care with the healthcare team. For persons with dementia, it is crucial to timely engage in ACP. Therefore, we study ACP in dementia using electronic health record data. This study aims to determine how often ACP conversations are recorded, analyze time from dementia diagnosis until the first recorded conversation and time from the first recorded conversation to death, and analyze which factors are associated with the timing of ACP.

**Methods:**

Electronic records of 15,493 persons with dementia in Dutch general practice between 2008 and 2016 were linked to national administrative databases. ACP conversations and indicators of health deficits to determine frailty were obtained from electronic records coded with the International Classification of Primary Care. Socio-demographic characteristics were derived from the national population registry managed by Statistics Netherlands. Date of death was derived from the Personal Records Database (2008–2018).

**Results:**

ACP was recorded as such as 22 (95% CI, 20–23) first conversations per 1,000 person-years of follow-up. The hazard ratio (HR) for the first conversation increased every year after dementia diagnosis, from 0.01 in the first year to 0.07 in the 7th and 8th year after diagnosis. Median time from a first conversation to death was 2.57 years (95% CI, 2.31–2.82). Migrant status [non-Western vs. Western (HR 0.31, 95% CI, 0.15–0.65)] was significantly associated with a longer time from dementia diagnosis to the first conversation. Being pre-frail (HR 2.06, 95% CI, 1.58–2.69) or frail (HR 1.40, 95% CI, 1.13–1.73) vs. non-frail was significantly associated with a shorter time from dementia diagnosis to the first ACP conversation.

**Conclusion:**

ACP conversations in Dutch general practice were rare for persons with dementia, or was rarely recorded as such. In particular among persons with a non-Western migration background and those who are non-frail, it started long after diagnosis. We advise further research into public health and practical strategies to engage persons with dementia with a non-Western migration background and non-frail persons early in the disease trajectory in ACP.

## Introduction

Advance care planning (ACP) is an essential element of good dementia care, since dementia is a progressive disease leading to severe cognitive decline (1, 2). In advanced stages of the disease, persons with dementia are no longer able to express their wishes and preferences. ACP can be defined as an ongoing communication process and as a continuous, dynamic process of reflection and dialogue about preferences for future care, between patient and the healthcare team but also with those close to the patient who may continue the dialogue with the healthcare team if the patient can no longer be involved (2–4).

Experiences during the COVID-19 pandemic highlighted the importance of knowing the needs and wishes of a patient and taking them into consideration to prevent unwanted care or life-sustaining treatment (5). For persons with dementia, it is especially important to express the needs and wishes early in the disease trajectory when decisional capacity is still intact (2, 3, 6).

General practitioners (GPs) are in a key position to initiate ACP with persons with dementia, because persons with dementia and their family caregivers usually have long-lasting relationships with their GPs, GPs are usually involved early in the disease trajectory, and because of a gatekeeping role of general practice in some healthcare systems (7). Previous research, however, has shown that preferences for future care are often not discussed with persons with dementia or it takes place too late in the disease trajectory (3, 8–10). Both persons with dementia and GPs could initiate ACP, however persons with dementia and family caregivers may hesitate to bring up ACP, while GPs may wait with initiating ACP until the person with dementia declines (11). Persons with dementia and family caregivers can be reluctant to initiate ACP due to, for example, discussing death or dying or fear of death. Encouraging persons with dementia and family caregivers by, for example, normalizing sensitive subjects, could be essential to start ACP discussions (11, 12). It is therefore important that GPs offer ACP. Longitudinal data that allow for time estimates from dementia diagnosis to ACP and ACP to death, however, are sparse.

Various barriers to initiating ACP have been reported (12, 13). Difficulty in determining the right timing to initiate ACP is one such barrier and there is much ambiguity among physicians on whether ACP should be started at the time of diagnosis (13, 14). Studies have shown that compared to persons with a Western background, persons with a non-Western migration background have fewer ACP conversations with healthcare professionals, which may be due to a language barrier or different perspectives with regard to ACP (15–18). A review on factors associated with the initiation of ACP in dementia showed that a patient's health status was an important factor, and that ethnic minority status may be a barrier to initiating ACP (12).

Data on characteristics of persons with dementia who are and are not engaged in ACP timely could inform targeted strategies to encourage ACP. Linking electronic health record data from GPs with national administrative data provides the opportunity to examine the occurrence and timing of ACP in longitudinal data of a representative large sample of persons with dementia to inform a public health approach or tailored approach to ACP in dementia. Using such data from Dutch national registries, this research aims to assess how often ACP was initiated after diagnosis, to estimate time from dementia diagnosis to the first recorded ACP conversation, and to estimate time from the first recorded ACP conversation to death. In addition, we aim to examine which characteristics, such as migrant status or frailty are associated with the timing of ACP in relation to dementia diagnosis and death.

## Materials and Methods

We used electronic health record (EHR) data from general practices in the Netherlands to select persons with a dementia diagnosis and to examine whether and when an ACP conversation took place during the disease trajectory. Their data were linked with national administrative databases to include socio-demographic characteristics and examine whether and when they died. Frailty was derived from the EHRs.

### Data Sources

In the Netherlands, almost all Dutch citizens are registered with a general practice. The GP acts as the gatekeeper to specialist care and is usually the first healthcare professional to contact with health problems, including cognitive problems (19). The diagnosis can be made by the GP or through referral to a specialist (20). After dementia has been diagnosed, the diagnosis will be recorded in the EHR system of GPs.

For this study, the EHRs from Dutch GPs who participated in the NIVEL Primary Care Database (NIVEL-PCD) between 2008 and 2016 were used to select persons with a dementia diagnosis, to examine recordings of ACP conversations, and to derive a frailty index score (21, 22). Practices can register at NIVEL to participate in this data collection. This database provides pseudonymized data from 451 general practices and covers approximately 10% of the Dutch population. It is representative for Dutch family practices in terms of age, sex, practice size, and geographical distribution of patients. GPs receive feedback on the quality of recording and are supported in coding (23). Additionally, there is a financial incentive as the GPs' reimbursement is based in part on the quality of the recording (24). Dementia diagnosis and ACP are entered in the EHR system and coded with ICPC-1 (International Classification of Primary Care) (25). Dementia diagnosis is coded under ICPC code P70 and ACP is generally coded under ICPC code A20.

To determine the time from the first recorded ACP conversation to death, the date of death was derived from the Municipal Personal Records Database (2008–2018) made available for research purposes by Statistics Netherlands. Patient socio-demographic characteristics (age, gender, migrant status, and living situation) at the time of dementia diagnosis were derived from the national population registry managed by Statistics Netherlands, as not all this information is available in the EHR.

### Study Population

**Figure 1:**
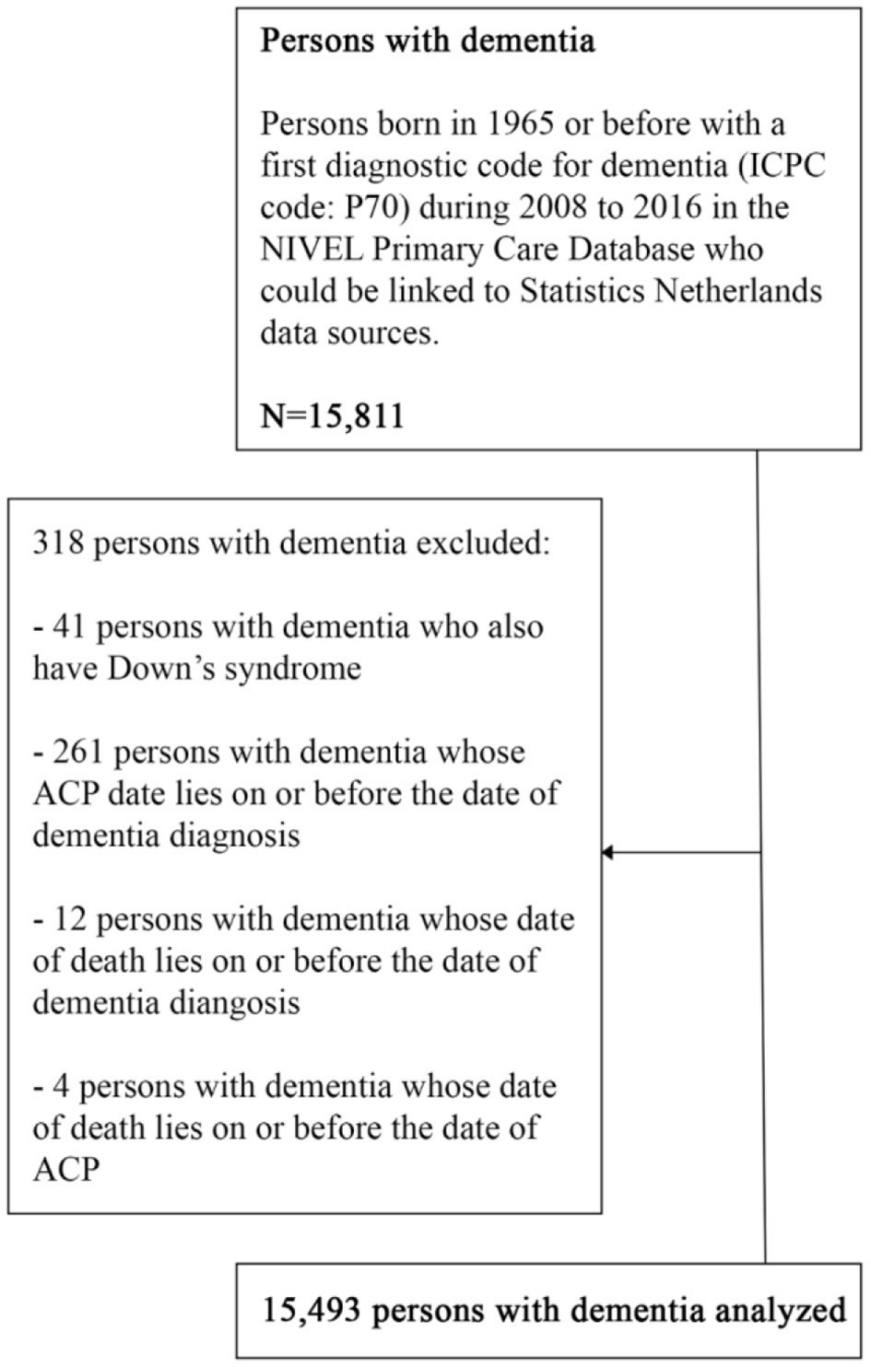
Flow diagram of the selection of the study sample.

Persons with dementia born in 1965 or before with a recorded dementia diagnosis between 2008 and 2016 and under the care of the GP at the date of diagnosis were included. Persons with dementia and Down syndrome were excluded, because of different care trajectories for these persons. We also excluded 277 persons where the date of ACP was recorded on or before the date of dementia diagnosis or where the date of death was recorded on or before the date of dementia diagnosis or ACP. These exclusions concerned <2% of all persons with dementia (Figure 1).

### Outcomes

Outcome measures included (1) the number of first ACP conversations regarding persons with dementia per 1,000 person-years of follow-up after the dementia diagnosis was recorded; (2) time from the recorded dementia diagnosis to the first recorded ACP conversation in the EHR; (3) time from the first recorded ACP conversation to death; (4) and associations between socio-demographic characteristics and frailty with the timing of ACP.

Before 2018, there was no clear ICPC code to record an ACP conversation in a patient's medical record. GPs were therefore advised to record ACP conversations under ICPC code A20 (26, 27). The standard ICPC label of A20 was “request/conversation about euthanasia”. Since euthanasia can be discussed during an ACP conversation, the Dutch College of General Practitioners regarded A20 as the best available option to record all ACP conversations, including those where euthanasia was not discussed (26, 27). In 2014, Ott and colleagues advised GPs to use A20 for recording wishes and decisions made during the ACP conversation, and to use ICPC code A58 (labeled as “ACP”) for recording an exploratory conversation about ACP (28). In 2017, Ott et al. mention A20 only for recording ACP conversations in a guideline for ACP in general practice (28). The analyses were limited to documented ACP wishes and decisions coded under A20.

### Socio-Demographics and Frailty

Sociodemographic information was derived at the time of the recorded dementia diagnosis. Migrant status was categorized as non-Western migration background (combining Surinamese, Antillean, Aruban, Moroccan, Turkish, or other non-Western migration background) and Western background (a native Dutch background or Western migration background).

Other variables were categorized as follows: (1) Age under 65 years old, 65–74, 75–84, and 85 and above; and (2) living situation with one or more cohabitants, living alone, or living in an institution.

A frailty index was created by screening the GP EHRs for 35 predefined clinically relevant “health deficits” including ICPC codes of diseases and symptoms, and one deficit “polypharmacy” (29). Examples of the health deficits include: general complaints, hearing and visual impairment, respiratory problems, heart failure, vascular disease, diabetes mellitus, neurological disease, psychiatric problems and social problems. The number of deficits present in an individual relative to the total number of possible deficits resulted in the Frailty Index (FI) score, with a range from 0 to 1. Persons with dementia were classified into three categories in accordance with prior studies: non-frail (three or fewer deficits; FI score ≤ 0.08), pre-frail (four to eight deficits; 0.08 < FI score < 0.25), and frail (nine or more deficits; FI score ≥ 0.25) (30–33).

### Data Linkage

Once the data from the GPs' EHRs were pseudonymized, they were transferred to Statistics Netherlands. Data linkage was performed by Statistics Netherlands. The citizen service number or a combination of birth date, gender and zip code was used to create the pseudonyms. In total 91.1% of the data was successfully matched.

### Data Analysis

Descriptive statistics were used to describe the characteristics of the study sample. The rate of first ACP conversations was calculated per 1,000 person-years of follow-up “at risk” for a first conversation as the follow-up times of persons with dementia varied. This was estimated by dividing the total number of first ACP conversations of persons with dementia by the total number of person-years of the sample, multiplied by 1,000. The 95% confidence interval was calculated by the formula: survival estimate ±1.96 times the corresponding SE.

To examine the association between socio-demographic characteristics, frailty and the time from the first recorded ACP conversation to death, we used Cox proportional hazard models. Hazard ratios (HRs) and 95% confidence intervals (CIs) were calculated for each variable.

Because death alters the probability of engaging in ACP (persons who die before ACP cannot engage in ACP any longer), it was considered as a competing risk. The standard Cox proportional hazard regression produces biased results in the presence of a competing risk (34). Therefore, the cumulative incidence function, as part of the competing risk approach, was used to estimate the time from diagnosis to the first recorded ACP conversation. The endpoint was ACP, the discharge date or death. If an individual had not died or engaged in ACP, or was not discharged from general practice at the end of data collection, his or her time to ACP was censored at the end of the observation period.

We used Kaplan Meier survival analyses to estimate time to death after the first recorded ACP conversation. For the analysis of associations between socio-demographic characteristics, frailty and engaging in an ACP conversation, we performed competing risk regression analysis.

Significance level for all analyses was set at 0.05. Competing risk analyses were performed using R studio (R version 3.6.2), with use of package cmprsk. SPSS version 25 was used for the other analyses.

## Results

### Study Sample

In total, 15,811 persons with dementia were identified and 15,493 were included for analysis (Figure 1). The average age of the sample was 81 years, with 45.9% of the sample being between the ages of 75-84 (Table 1). Most persons with dementia (63.3%) were female. Most lived with cohabitants (46.3%), 41.4% lived alone and 12.2% lived in a long-term care facility (probably without 24/7 oversight as medical nursing home care is not provided by the GP). A large portion of the sample (87.3%) had a native Dutch background. Almost three quarters (72.4%) were classified as pre-frail.

**Table 1:** Characteristics of persons with dementia (*n* = 15,493).

	***N***	**%**
Female gender	9,805	63.3
Age, mean (SD)	81.16	(8.13)
Under 65	636	4.1
65–74	2,453	15.8
75–84	7,111	45.9
85 and above	5,293	34.2
Living situation		
With one or more cohabitants	7,181	46.3
Alone	6,408	41.4
In an institution	1,892	12.2
Migrant status		
Native Dutch	13,529	87.3
Western migration background	1,524	9.8
Surinamese/Antillean/Aruban	184	1.2
Moroccan/Turkish	176	1.1
Other non-Western	80	0.5
Frailty index (FI, 0-1), median (range)	0.14	(0.47)
Mean (SD)	0.14	(0.07)
Non-frail (FI ≤ 0.08)	2,578	16.6
Pre-frail (0.08 > FI > 0.25)	11,219	72.4
Frail (FI ≥ 0.25)	1,696	10.9

### Rate of First ACP Conversations and Number of ACP Conversations

Between 2008 and 2016, ACP was initiated 22 (95% CI, 20–23) times per 1,000 person-years of follow-up after the dementia diagnosis was recorded, as recorded in their GP record. In total, there were 801 persons who engaged in ACP conversations during 26,809 person years. Those who engaged in ACP conversations, were involved in one to six ACP conversations with their GP (Table 2), mostly (80.3%; 643/801) it concerned a single conversation.

**Table 2:** Number of ACP conversations conducted in the period 2008–2016.

**Number of ACP conversations per person**	**Persons with dementia (*n* = 15,493) *n (%)***
1	643 (4.2)
2	112 (0.7)
3	32 (0.2)
4–6	14 (0.1)
Total	801 (5.2)

### Time From Diagnosis to ACP and Time From ACP to Death

**Figure 2:**
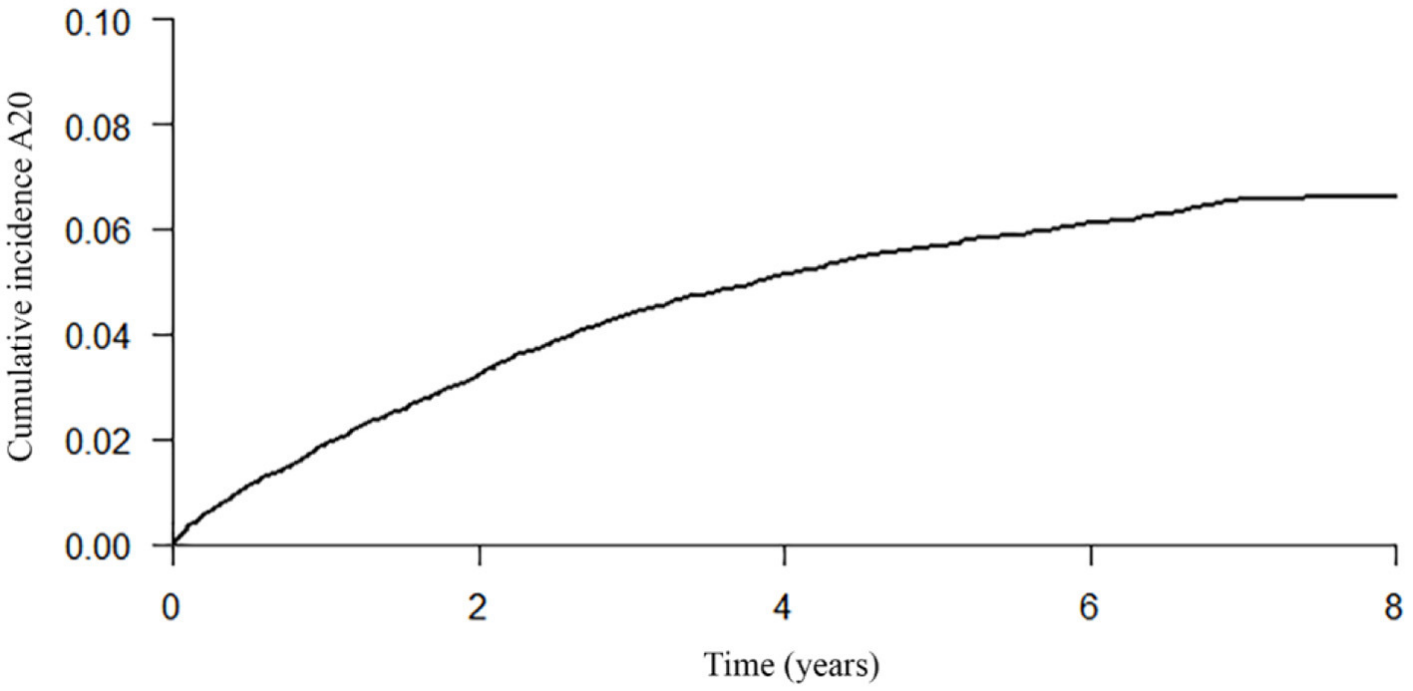
Cumulative incidence curve of a first recorded ACP conversation until 8 years after dementia diagnosis.

The probability of engaging in a first ACP conversation within 6 months up to 8 years after diagnosis was estimated and increased every year, starting with 0.01 in the first year after the dementia diagnosis was recorded and increasing to 0.07 in year 7 and 8 (Table 3; Figure 2), indicating that the probability of ACP initiated increased over the years but remained low. The median time from dementia diagnosis to a first recorded ACP conversation could not be estimated as less than half of the persons with dementia engaged in an ACP conversation at the end of the study period (therefore the cumulative incidence curve remained below 50% at the end of the study period).

**Table 3:** Probability of a first ACP conversation within 6 months up to 8 years after dementia diagnosis and probability to survive at least 6 months up to 7 years after ACP.

	Probability of ACP conversation^*^		Probability of surviving^*^	
	**Persons with dementia** **(*****n*** **=** **15,493)**		**Persons with dementia** **(*****n*** **=** **801)**	
**Years**	**Estimate**	**95% CI**	**Estimate**	**95% CI**
0.5	0.01	0.01; 0.01	0.95	0.92; 0.96
1	0.02	0.02; 0.02	0.73	0.70; 0.76
2	0.03	0.03; 0.04	0.58	0.54; 0.61
3	0.04	0.04; 0.05	0.44	0.40; 0.47
4	0.05	0.05; 0.06	0.33	0.30; 0.36
5	0.06	0.05; 0.06	0.25	0.21; 0.28
6	0.06	0.06; 0.07	0.20	0.16; 0.23
7	0.07	0.06; 0.07	0.12	0.07; 0.17
8	0.07	0.06; 0.07	–	–

* *The probability of ACP conversations were derived from the competing risk analysis. The probability of surviving were derived from the Kaplan Meier output*.

**Figure 3:**
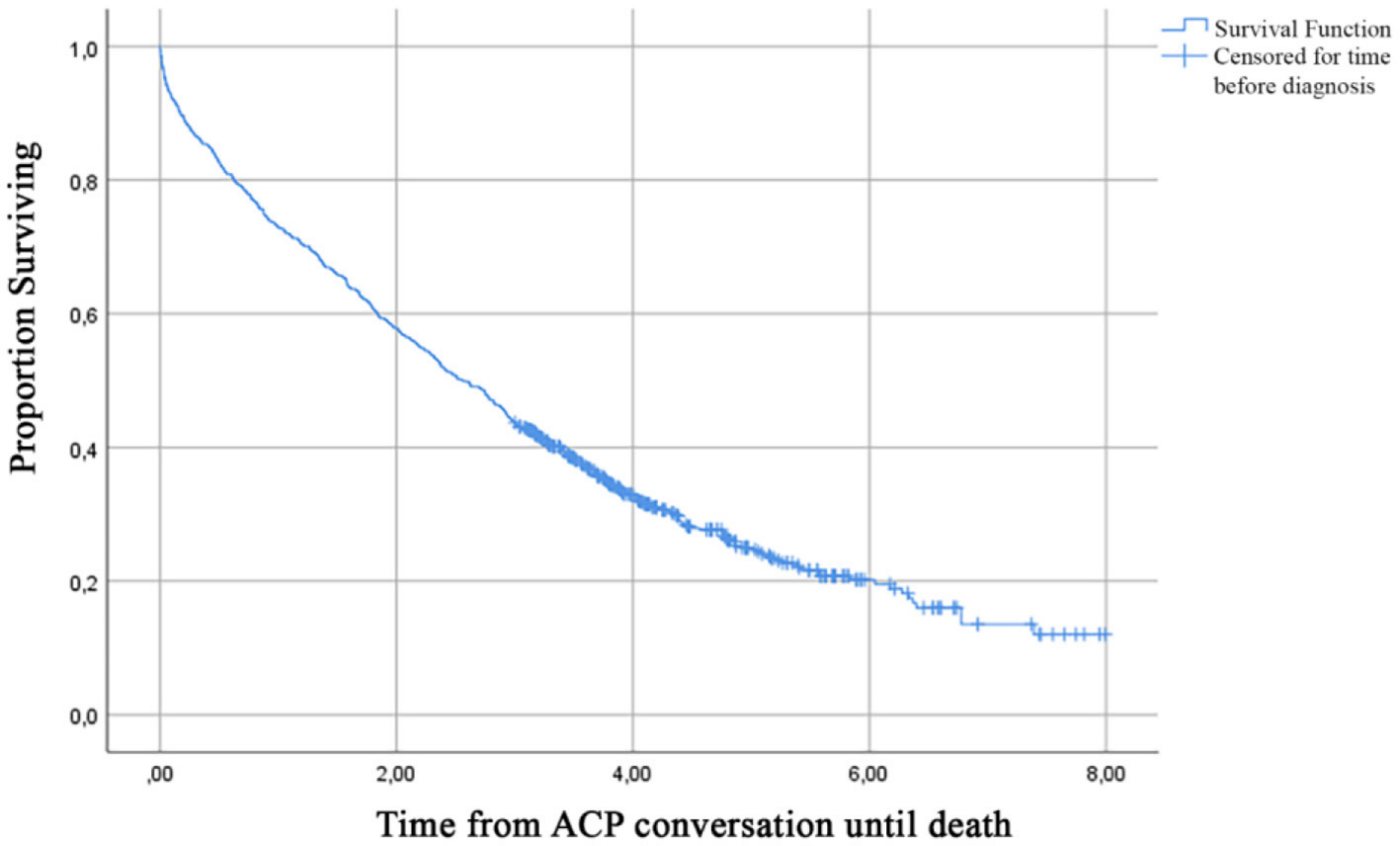
Kaplan Meier curve of time in years from a first recorded ACP conversation until death.

Median time from the first recorded ACP conversation to death was 2.57 years (95% CI, 2.31–2.80) (Figure 3). Table 3 presents the probability to survive at least 6 months up to 7 years after the first recorded ACP conversation. This probability to survive ranged from 0.95 in the first year to 0.12 at 7 years after ACP.

### Factors Associated With Time From Dementia Diagnosis to Engaging in ACP and With Time From ACP to Death

Non-Western migration background was significantly associated with a longer time from diagnosis to the first recorded ACP conversation while being pre-frail or frail was significantly associated with a shorter time to the first recorded ACP conversation compared to being non-frail (Table 4). Older age, male gender, living together with one or more cohabitants, and being frail were associated with a shorter time from the first recorded ACP conversation to death.

**Table 4:** Results of competing risk regression examining characteristics associated with time from diagnosis to a first ACP conversation, and results of Cox proportional hazard regression examining characteristics associated with time from a first ACP conversation after diagnosis to death in persons with dementia.

	**Time to first ACP**		**Time to death after first ACP**	
	**Persons with dementia (*****n*** **=** **15,493)**		**Persons with dementia (*****n*** **=** **801)**	
**Outcome**	HR^*^	**95% CI**	HR^*^	**95% CI**
Older age (65+)	0.81	0.58; 1.12	1.64^**^	1.08; 2.50
Age category (ref under 65)	Ref	Ref	Ref	Ref
65–74	0.84	0.58; 1.20	1.02	0.64; 1.63
75–84	0.78	0.56; 1.09	1.53	1.00; 2.35
85 and above	0.84	0.60; 1.18	2.53^**^	1.64; 3.90
Female gender	1.03	0.89; 1.19	0.64^**^	0.54; 0.77
Living situation (ref living together with 1 or more cohabitants)	Ref	Ref	Ref	Ref
Living alone	0.96	0.82; 1.12	0.67^**^	0.51; 0.89
Living in an institution	0.87	0.69; 1.11	0.67^**^	0.52; 0.88
Non-Western (vs. Native Dutch or Western migration background)	0.31^**^	0.15; 0.65	1.19	0.44; 3.18
Frailty index (ref non-frail)	Ref	Ref	Ref	Ref
Pre-frail	2.06^**^	1.58; 2.69	1.35	1.03; 1.77
Frail	1.40^**^	1.13; 1.73	1.56^**^	1.12; 2.16

* *Hazard ratios (HRs) are adjusted for age and gender. HR > 1 indicates shorter time to first ACP or death. HR < 1 indicates longer time to first ACP or death*.

** *P-value < 0.05*.

## Discussion

### Main Findings

Our study showed that ACP was initiated 22 times per 1,000 person-years of follow-up in persons with dementia in the Netherlands, as recorded in their GP record. In half of the cases, the first ACP conversation occurred within 2.57 years before death.

### ACP Prevalence and Time to ACP and Death

Although we estimated the rate of first ACP conversations per 1,000 person-years, which is not directly comparable to a percentage, this figure is clearly low compared to an ACP prevalence of 34% GPs reported in terminally ill patients in general practice in the Netherlands and Belgium (35). It is also much lower than the prevalence of any ACP reported after death of nursing home residents with dementia in Belgium (11.8%) (36).

The percentage of 34, however, referred to ACP which included agreements about care made with family only in a terminally ill population with non-sudden unexpected deaths according to their GP, and almost half died from malignancies. ACP in an unselected population of persons with dementia is not expected to occur equally often. Nevertheless, the differences can also be due to missing ACP conversations in this study as we identified ACP conversations based on GPs' recordings of “request/conversation about euthanasia” (ICPC A20). It could be that some GPs choose to record conversations that contained ACP elements under different ICPC codes than the recommended code. Further, probably not all ACP conversations were recorded consistently due to different conceptualizations of ACP among GPs (37).

In addition, the label of A20 and the advice on coding before 2018 denote a more medically oriented approach to ACP or even a belief that euthanasia requests of the person with dementia themselves are worthy of recording while other conversations less so. Perhaps the ACP conversations that have been coded were more focused on detailed advance medical treatment orders. ACP, however, can also be approached differently, focused on global goal setting based on what persons find important in life (38).

Based on clinical experience, we believe that more informal ACP conversations take place which are not documented. Recording ACP conversations may also happen in other ways other than documented with ICPC code A20, for example, it can be put in a memo; a note in the medical file. The experience is also that with new ICPC codes, it may take a while before they have been implemented well. In addition, ACP discussions about care with the family caregiver, for example, may also not be documented in the patient record, but narratives may be shared amongst colleagues at the general practice during work meetings.

It is also possible that ACP conversations occurred later, after admission to the nursing homes. These are not recorded in the EHRs, as persons with dementia in nursing homes do not receive care from their GPs anymore.

One study showed that compared to cancer patients and patients with organ failure, patients with dementia engaged in ACP less often (10). For example, a surrogate decision-maker was appointed in only 13 of 73 persons with dementia, and end-of-life-treatment preferences were known of 28 of 73 persons with dementia (10). Therefore, our and other studies suggest that ACP occurs infrequently in particular in persons with dementia in general practice. This may be due to various identified barriers to initiating ACP by the GP, the person with dementia or family caregiver, such as identifying the right time to start ACP, worries that ACP might cause stress or fear with the person with dementia or no awareness of the importance of ACP by the family caregiver until it was too late (13, 37, 38).

A study on end-of-life treatment decisions for persons with dementia found that advance directives were rare: 4.9% (16 of 325 persons with dementia) had completed an advance directive before nursing home admission (39). This further supports our finding of the low number of recorded ACP.

Regarding time from dementia diagnosis to the first recorded ACP conversation, we found that with every year after diagnosis, the probability of ACP being initiated increased but remained low. At year 7 and 8 the hazard ratio for a first ACP conversation remained 0.07. A qualitative study showed that some GPs, persons with dementia and family caregivers preferred to discuss preferences for the future once problems arise, which could mean later in the disease trajectory (7). This is also in line with our result that initiating ACP is slightly more likely to occur as the years pass.

### Patient Groups With Different Time to ACP

Our study showed that persons with dementia with a Western background engaged in an ACP conversation sooner after dementia diagnosis than those with a non-Western migration background. A systematic review on ACP in dementia also found that ethnic minority status was associated with a lower chance of initiating ACP (12). Most of the studies included in the systematic review were from the US. Other studies from the US show that persons with a non-Western migration background or ethnic minority status are less likely to engage in ACP conversations with healthcare professionals than persons with a Western background (15–17).

Studies carried out in the US, however, concern patients with different migration backgrounds than those in the Netherlands who were included in our study. A qualitative study with Turkish and Moroccan patients on palliative care showed that there are several specific barriers for persons with these migration backgrounds to initiate discussions on palliative care (18). Examples are a language barrier (such as lack of proper translation of jargon used), taboo to talk about difficult topics, and lack of separate discussions with family caregivers. These reasons may partly explain our finding of a longer time from diagnosis to engage in ACP in persons with dementia with a non-Western migration background. Other possible barriers may be differences in perspectives with regard to ACP, and possibly a lack of tools for GPs to initiate ACP with persons with a non-Western migration background. We therefore recommend further studies on the perspectives regarding ACP of persons with dementia with a non-Western migration background, their family caregivers and GPs, and on the development of accessible tools that could facilitate ACP.

Another factor we found to be associated with a shorter time to engaging in the first ACP conversation after dementia diagnosis was being pre-frail or frail. Being pre-frail indicates four to eight out of 36 (health) deficits, and being frail with nine or more health deficits. One study showed that some GPs would initiate ACP once cognitive deterioration has become problematic, which may mean that deterioration of the health condition is one of the triggers to initiate ACP (7). Therefore, being pre-frail or frail can indicate worsening of the health condition and may trigger ACP. While it is advised to initiate ACP before health deteriorates, our study results indicate the opposite occurs (40). Other studies found that the condition of the person with dementia was a factor that influenced ACP initiation (41, 42). This suggests a more reactive approach of the GP to ACP, rather than a proactive approach (41). We recommend integration of ACP and palliative care in holistic interventions that could ensure early initiation of ACP and goal concordant care once the health condition worsens (43).

Regarding frailty, our results additionally showed that compared to non-frail being pre-frail was associated with a shorter time to engage in ACP than being frail. An explanation could be that persons with dementia who are frail may be further in the disease trajectory which could act as a barrier to initiate ACP by either the person with dementia, family caregiver or healthcare professional.

### Strengths and Limitations

To our knowledge, this study is the first that examined the occurrence of ACP conversations in primary care practice after a dementia diagnosis is recorded and examined its timing relative to diagnosis and death. Other studies, mostly conducted in English-speaking countries and in long-term care facilities, usually do not report on ACP occurrence over time, or are limited to a single community healthcare center [e.g., (44)].

The findings concerned only persons with dementia with a recorded diagnosis of dementia. An important limitation regarding occurrence of ACP conversations is a probable underestimation as not all ACP conversations might have been recorded under ICPC code A20. In addition, probably not all informal ACP conversations were recorded. Further, we did not capture discussions about care preferences in non-health care settings while such discussions with persons with dementia may be more frequent than with healthcare professionals (45). In addition, no good-quality information about capacity or severity of dementia is collected routinely in practice; therefore this was not available from the EHR, while this might influence the initiation of ACP. We may also have missed dementia cases. There may be delay between initial dementia symptoms and the recording of a dementia diagnosis (46). There is underreporting of dementia diagnoses in patient records, especially of persons with mild dementia (47–50). A qualitative study with family caregivers about the timing of diagnosis in five European countries showed that 47.1% of family caregivers (36.5% Dutch family caregivers) thought that it would have been better if the dementia diagnosis had been made earlier (51). A survey across Europe showed that GPs agree that diagnosis of Alzheimer's disease is often delayed (52). Underdiagnosis of dementia also occurs in persons with a migration background. Therefore, we may also have missed more persons with a migration background who have dementia (53, 54). We do not know whether we are underestimating or overestimating ACP for persons with dementia by missing dementia cases. We were limited in examinating of factors potentially associated with the outcomes and we did not use time-varying covariates. However, using covariates at the time of dementia diagnosis makes more sense in relation to our objectives. This way, we can better understand patient factors at the time of diagnosis that drive initiation of ACP, which is clinically relevant to ACP conducted early in the disease trajectory.

## Conclusion

Our study showed a low number of recorded ACP conversations in general practice among persons with dementia and few persons having any. A non-Western migration background was significantly associated with a longer time to newly engage in ACP after diagnosis, while being pre-frail or frail was associated with a shorter time to engaging in ACP, indicating that ACP might not occur early in the disease trajectory. We advise further research into public health and practical strategies to engage persons with dementia with a non-Western migration background in ACP as well as persons with dementia early in the disease trajectory who are non-frail. We also recommend qualitative research to find out whether all ACP conversations can be captured from electronic health records to determine how much of formal and more informal conversations is actually documented in the health record, and study within-family conversations and prepare family and patients for ACP discussions with educational materials to encourage them to raise topics of their choice (55, 56). Last, we recommend consistent reporting of ACP conversations with specific formats, such as a form that can be used to record ACP conversations. This will also support accuracy of ACP incidence figures in future cross-national comparisons when such data become increasingly available from more nations.

## Data Availability Statement

The datasets presented in this article are not readily available because the datasets used for this study are not publicly available, as these are stored within the safe environment of Statistics Netherlands and cannot leave this environment according to their safety conditions. Requests to access the datasets should be directed to the principal investigator of the BESIDE study (k.joling@amsterdamumc.nl)

## Ethics Statement

The studies involving human participants were reviewed and approved by the Medical Ethical Committee of the VU University Medical Center. Written informed consent for participation was not required for this study in accordance with the national legislation and the institutional requirements.

## Author Contributions

KJ performed the data cleaning and preparation. BA and KJ analyzed the data. BA, KJ, JS, and BT interpreted the data. BA prepared an initial version of the manuscript for submission. KJ and JS supervised the work and contributed equally. All authors contributed to the article and approved the submitted version.

## Funding

The work was supported by the Netherlands Organization for Health Research and Development (ZonMw), Grant Number is 733050403 and the European Research Council, Grant Agreement ID: 771483.

## Conflict of Interest

The authors declare that the research was conducted in the absence of any commercial or financial relationships that could be construed as a potential conflict of interest. The reviewer FP is currently organizing a Research Topic with one author HH.

## Publisher's Note

All claims expressed in this article are solely those of the authors and do not necessarily represent those of their affiliated organizations, or those of the publisher, the editors and the reviewers. Any product that may be evaluated in this article, or claim that may be made by its manufacturer, is not guaranteed or endorsed by the publisher.
